# Individual Variation in Conditional β Cell Ablation Mice Contributes Significant Biases in Evaluating β Cell Functional Recovery

**DOI:** 10.3389/fendo.2017.00242

**Published:** 2017-09-14

**Authors:** Song Lu, Jiatao Li, Kathy O. Lui

**Affiliations:** ^1^Li Ka Shing Institute of Health Sciences, The Chinese University of Hong Kong, Prince of Wales Hospital, Hong Kong, Hong Kong; ^2^Department of Chemical Pathology, The Chinese University of Hong Kong, Prince of Wales Hospital, Hong Kong, Hong Kong

**Keywords:** VEGF, modified mRNA, pancreatic beta cells, regeneration, diabetes mellitus, type 1

## Abstract

Despite the βDTA (Ins2-rtTA; Tet-DTA) mice have been developed as a valuable tool to study β cell regeneration, their individual variation in therapeutic efficacy has not been characterized. Here, we demonstrated that the βDTA mice exhibited significant variations in both spontaneous and acquired β cell regeneration. We found that doxycycline (DOX)-induced β cell death was sufficient to cause polydipsia, translating even subtle difference in drinking habit into large variations in actual DOX intake among individuals within the same group. Accumulating evidence shows that transient expression of VEGF_A_ enhances β cell functional recovery after injury. Therefore, we utilized the chemically modified mRNA (modRNA) technology to enable transient yet efficient VEGF_A_ expression in the pancreas after DOX-induced β cell death. Surprisingly, under optimized DOX dose permissive of β cell regeneration, VEGF_A_ modRNA only demonstrated marginal benefits on β cell functional recovery with large individual variations. We also revealed that the therapeutic efficacy of VEGF_A_ modRNA on β cell regeneration was dependent on the degree of β cell loss induced by the accumulated DOX intake. Therefore, our results highlight a significant contribution of individual variation in the βDTA model and call for attention in evaluating potential efficacy of therapeutic agents in β cell regeneration studies.

## Introduction

Pancreatic islets are highly vascularized; pancreatic β cells and microvascular endothelial cells (ECs) have an interdependent physical and functional relationship (1). In an adult pancreas, β cells secrete angiogenic factors such as VEGF_A_ to maintain survival and growth of the adjacent pancreatic ECs that facilitate efficient glucose sensing by and transport of secreted insulin from β cells (1, 2). Moreover, membrane proteins, such as laminins (3, 4), collagen (5) and integrins (4, 5) of the vascular basement membrane between pancreatic ECs and β cells, have been shown to stimulate β cell replication and insulin gene expression. During β cell injury such as in type-1 diabetes, it has been shown that EC regeneration is necessary to reverse hyperglycemia if autoimmunity is blocked (6). Taken together, accumulating evidence suggests that ECs support development, survival, function, and regeneration of pancreatic β cells. Harnessing EC-mediated β cell regeneration may shed light on development of novel therapeutics for treatment of diabetes.

More recently, it has been shown that VEGF_A_, an angiogenic factor that supports EC growth and function, is particularly important for pancreatic β cell development. Pancreatic progenitor specific knockout of VEGF_A_ in the Pdx1-Cre;Vegfa^fl/fl^ mice contributes to impaired glucose tolerance as a result of β cell hypoplasia in both developing and adult islets (7). The reduction in β cell mass of the Pdx1-Cre;Vegfa^fl/fl^ mice is not caused by reduced progenitor differentiation but reduced replication of preexisting β cells (7), which is the major mechanism by which β cells regenerate (8–10). Nevertheless, it has been shown that sustained overexpression of β cell-specific VEGF_A_ leads to β cell apoptosis and impaired glucose tolerance (11, 12); while only transient overexpression of β cell-specific VEGF_A_ induces β cell replication in adult mice (12, 13). Therefore, VEGF_A_ functions in a narrow physiological range to maintain islet homeostasis and function.

We have previously demonstrated that the modified mRNA (modRNA) technology has some unique advantages over traditional genetic manipulation methods (14, 15). We have demonstrated both *in vitro* (15) and *in vivo* (16) that VEGF_A_ modRNA directs cell fate decision of cardiovascular progenitors and promotes vascular regeneration in adult mouse heart after myocardial infarction (16). Therefore, here, transient yet controlled and highly efficient protein expression *via* modRNA offers a promising platform to examine the therapeutic potential of VEGF_A_ in β cell regeneration. We also employed the Ins-rtTA; TET-DTA mice as a β cell death model for our study. In fact, the TET-rtTA system has been widely used in transgenic models to allow reversible gene expression. Specifically, the Ins-rtTA; TET-DTA (βDTA) line was developed to induce β cell-specific cell death following doxycycline (DOX) administration to activate the diphtheria toxin A (DTA). Since DTA expression is turned off after DOX withdrawal, spontaneous β cell regeneration takes place given there is sufficient β cells remained (17). Similar to other transgenic lines with the TET-rtTA system, it is efficient and convenient to induce DTA expression by feeding the βDTA mice with DOX-containing drinking water (17–21).

In this study, we revealed that the βDTA mice exhibited significant variations in both spontaneous and acquired β cell regeneration. Such variations could be explained, at least in part, by the subtle difference in individual’s drinking habit and, therefore, DOX intake within the same group, amplified by a positive feedback loop between β cell loss and polydipsia. Furthermore, we also demonstrated that the therapeutic efficacy of VEGF_A_ modRNA in promoting β cell regeneration was sensitive to the initial degree of β cell loss as a result of internal variations in individual’s drinking habit. Since underestimating the internal variations within groups would contribute to biased interpretations as well as the difficulty in reproducing results from β cell regeneration studies (10), our study emphasized the importance of evaluating individual variations in response to therapeutic agents during β cell regeneration; and addressed the potential source of internal variations using the βDTA model.

## Results

### The Degree of Impaired β Cell Function Is Positively Correlated to the Amount of Accumulated DOX Intake in βDTA Mice

**Figure 1:**
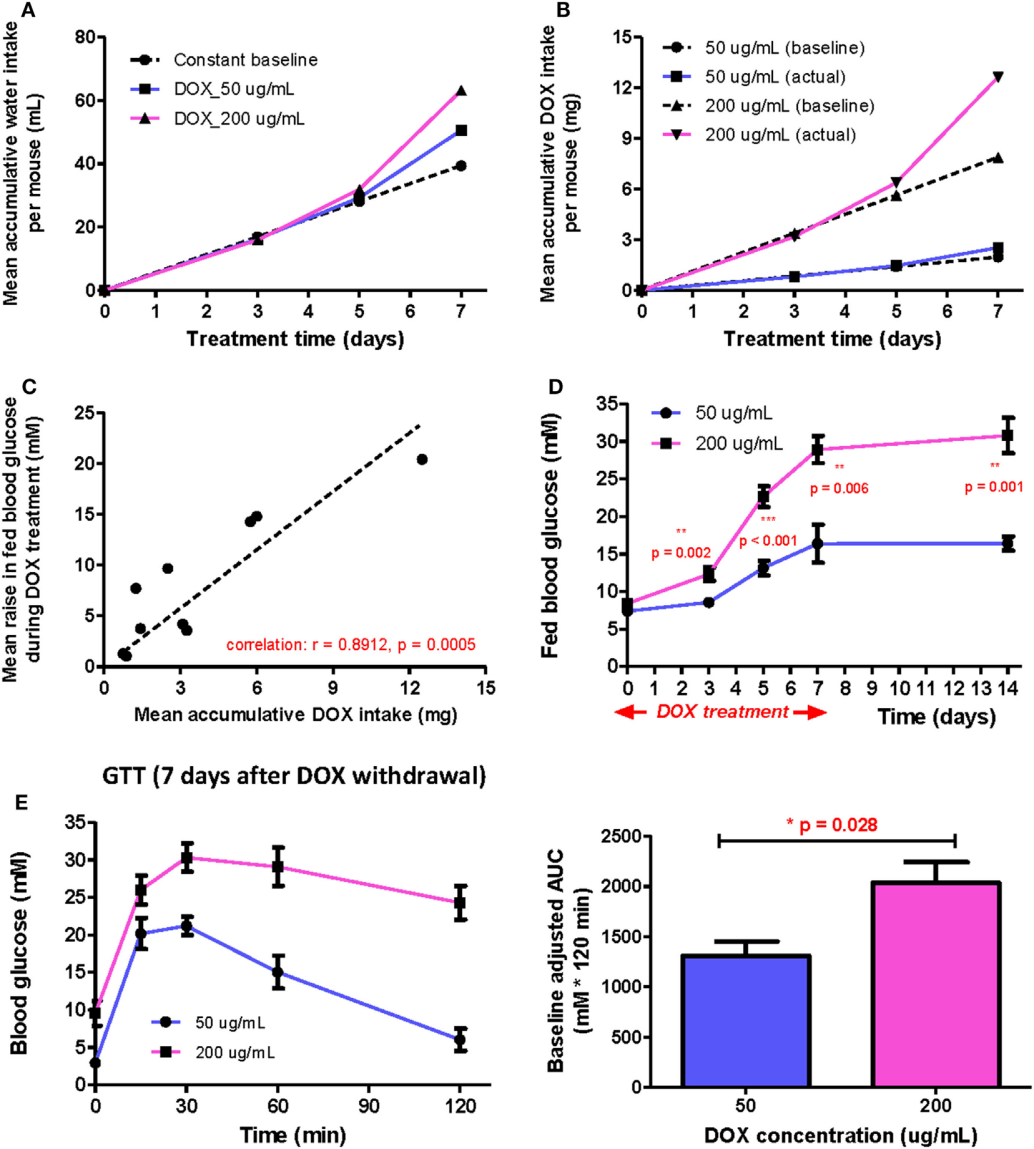
The positive correlation between β cell lesion and accumulative doxycycline (DOX) dose in βDTA mice during and shortly after DOX treatment. **(A)** Actual mean accumulative water consumption per mouse (solid lines) deviated from expected value (dashed line) based on constant baseline daily water consumption during DOX treatment. **(B)** The difference between actual and expected mean accumulative DOX intake per mouse for groups treated by different doses. **(C)** Positive correlation between the mean increase in fed glucose and mean accumulative DOX dose per mouse during treatment (Pearson correlation coefficient *r* = 0.8912, *p* = 0.0005, linear regression as the dashed line across origin). **(D)** Comparison of fed glucose between mice treated with 50 and 200 µg/mL DOX during treatment and 7 days after withdrawal (**p < 0.01, ***p < 0.001 by independent *t*-test comparing two groups at each time point). **(E)** Glucose tolerance test (GTT) (i.p.) at D7 after DOX withdrawal and comparison of baseline adjusted area under curve between two groups (*p = 0.028 by independent *t*-test). Values are mean ± SEM, *n* = 4 per group **(D,E)**; each data point represents mean value of four individuals **(A–C)**.

When DOX is administrated to mice *via* drinking water, the total DOX intake is in principle directly proportional to the DOX concentration and average daily water consumption before treatment. However, the drinking behavior of mice may vary during the indicated treatment period because DOX gradually induced hyperglycemia. To test this, we measured the mean accumulated water intake (Figure 1A) and dosage consumed (Figure 1B) for 1 week following treatment of 50 and 200 µg/mL DOX, respectively. We found that the mean volume intake and dosage consumed were higher in the DOX-treated groups (both 50 and 200 µg/mL) than the estimated rate (baseline) since day 5. We also found that the mean volume intake and dosage consumed increased more rapidly in the 200 than 50 µg/mL group compared to their respective baseline, indicating that polydipsia was more severe in the 200 than 50 µg/mL group. Our results suggest that polydipsia developed during the course of treatment possibly increased the daily water consumption leading to an increased DOX intake.

Although polydipsia caused a positive feedback in water and, therefore, DOX intake, there was still a strong positive correlation (*r* = 0.8912, *p* = 0.0005) between the increased fed glucose level and the accumulated DOX intake at any time point for both groups (Figure 1C). Moreover, the 200 µg/mL group also demonstrated a significantly higher mean fed glucose level than the 50 µg/mL group at the indicated time points even at 7 days post DOX withdrawal (Figure 1D). We also performed glucose tolerance test (GTT) at day 7 post DOX withdrawal to confirm a significantly more impaired glucose tolerance in the 200 than 50 µg/mL group (Figure 1E). Altogether, our results show that polydipsia developed during the course of treatment led to discrepancy between actual and expected DOX intake based on stable daily water consumption; but the degree of impaired β cell function was still correlated to the total DOX intake in a dose-dependent manner.

### 50 µg/mL DOX in Drinking Water Is Sufficient to Induce Beta Cell Loss while Retaining Their Regenerative Capacity in βDTA Mice

**Figure 2:**
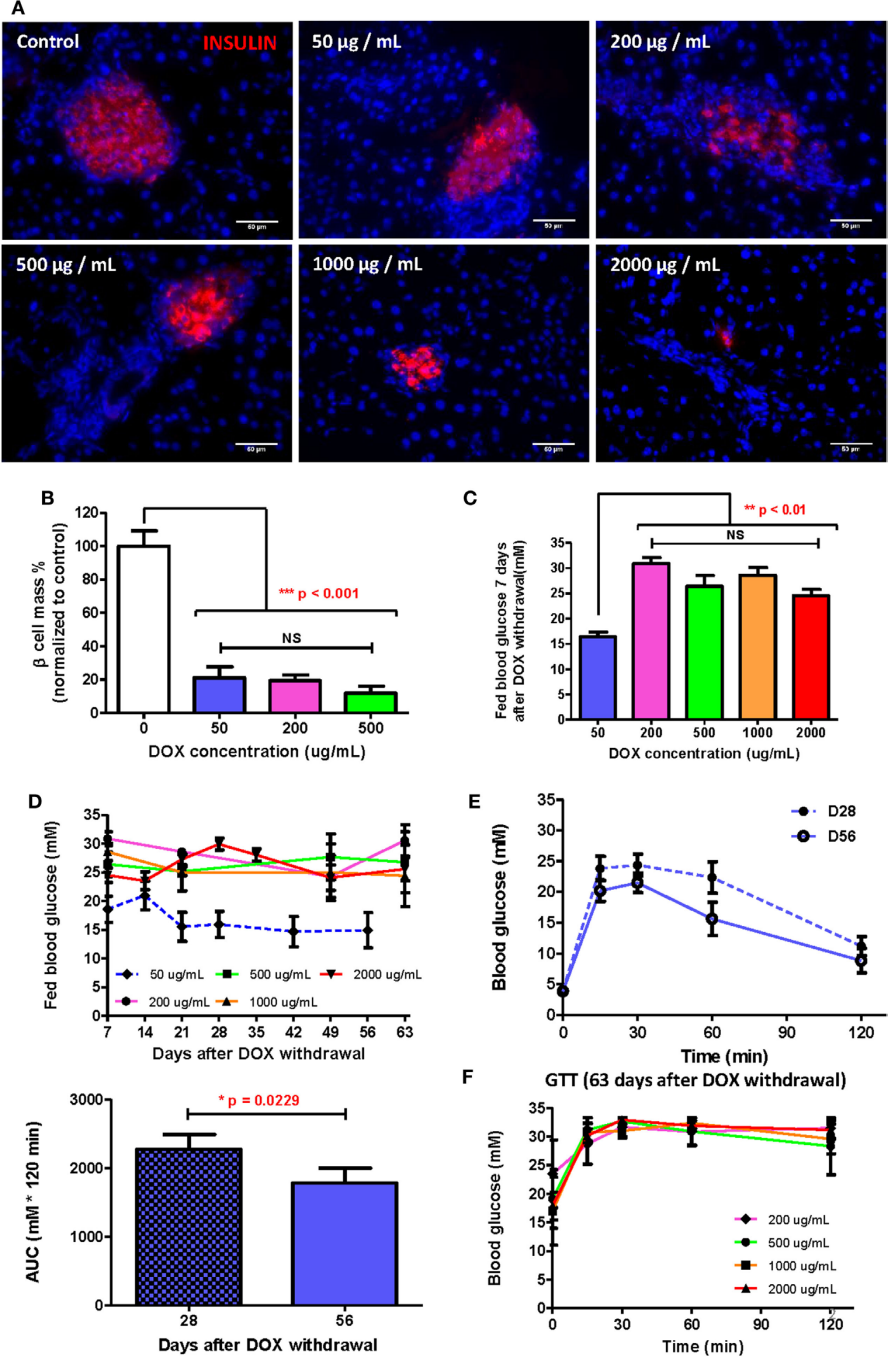
Doxycycline (DOX) dose optimization to allow β cell functional recovery within reasonable time. **(A)** Presentative insulin staining (red) in islets of mice treated by different DOX doses 7 days after withdrawal (bar, 50 µm). **(B)** Comparison of % β cell mass (normalized to the mean of control group) of different groups 7 days after DOX withdrawal (***p < 0.001, one-way ANOVA followed by Bonferroni’s multiple comparison test, *n* = 5 for 200 µg/mL group, *n* = 3 for other groups). **(C)** Comparison of fed glucose of different groups 7 days after DOX withdrawal (**p < 0.01, one-way ANOVA followed by Dunnett’s multiple comparison test with 50 µg/mL group as control, *n* = 4 for 50 µg/mL, *n* = 12 for 2,000 µg/mL, *n* = 7 for other groups). **(D)** Long-term fed glucose monitoring for different groups after DOX withdrawal (*n* = 12 for 2,000 µg/mL group, *n* = 10 for 50 µg/mL group, *n* = 4 for other groups). **(E)** Glucose tolerance test (GTT) (i.p.) and area under curve comparison at D28 and D56 for mice treated with 50 µg/mL DOX (*p = 0.0229 by paired *t*-test, *n* = 10 per time point). **(F)** GTT (i.p.) at D63 after DOX withdrawal for groups treated by 200–2,000 µg/mL DOX (*n* = 12 for 2,000 µg/mL group, *n* = 4 for other groups). Values are mean ± SEM.

Since adult β cells regenerate by replication from preexisting β cells (17, 21), severe β cell death could delay or inhibit β cell regeneration after injury. To determine the optimal dose of DOX treatment in long-term β cell regeneration studies, DOX was administrated in drinking water at 50, 200, 500, 1,000, and 2,000 µg/mL, respectively, for 7 days to induce β cell lesion in βDTA mice. At D7 posttreatment, immunostaining for INSULIN was performed (Figure 2A), and we found that the β cell mass was significantly reduced by at least 80% in all doses compared to the untreated control (Figure 2B). Although the size of individual pancreatic islets appeared to be smaller in the higher dosage groups (200–2,000 µg/mL, Figure 2A), there were more numbers of small islets in the higher dosage groups, so the overall β cell mass did not show significant difference in the range of 50–500 µg/mL (Figure 2B).

Interestingly, only the 50 µg/mL group showed a significantly lower fed glucose level than the higher dosage groups; whereas the higher dosage groups showed similar high fed glucose levels at D7 posttreatment (Figure 2C). A similar trend was also observed in long-term monitoring of fed glucose level, in which the 50 µg/mL group demonstrated recovery at D21 posttreatment with fed glucose remained stable afterward; whereas the higher dosage groups showed no decline in fed glucose levels (Figure 2D). Our GTT data also confirmed that the 50 µg/mL group demonstrated a continued improvement in glucose tolerance from D28 to D56 posttreatment (Figure 2E); whereas the higher dosage groups revealed sustained impaired glucose tolerance at D63 posttreatment (Figure 2F). In fact, the glycemic control in the higher dosage groups was so impaired that even the fasting glucose levels remained elevated for 63 days (Figure S1 in Supplementary Material). Therefore, a treatment of 50 µg/mL DOX for 7 days was sufficient to induce significant β cell loss yet retain their regenerative capability. Our results also indicated that well-preserved β cell morphology was essential for β cell regeneration.

### A Single Dose of Human VEGF_A_ (hVEGF_A_) modRNA Induces β Cell Regeneration in βDTA Mice

**Figure 3:**
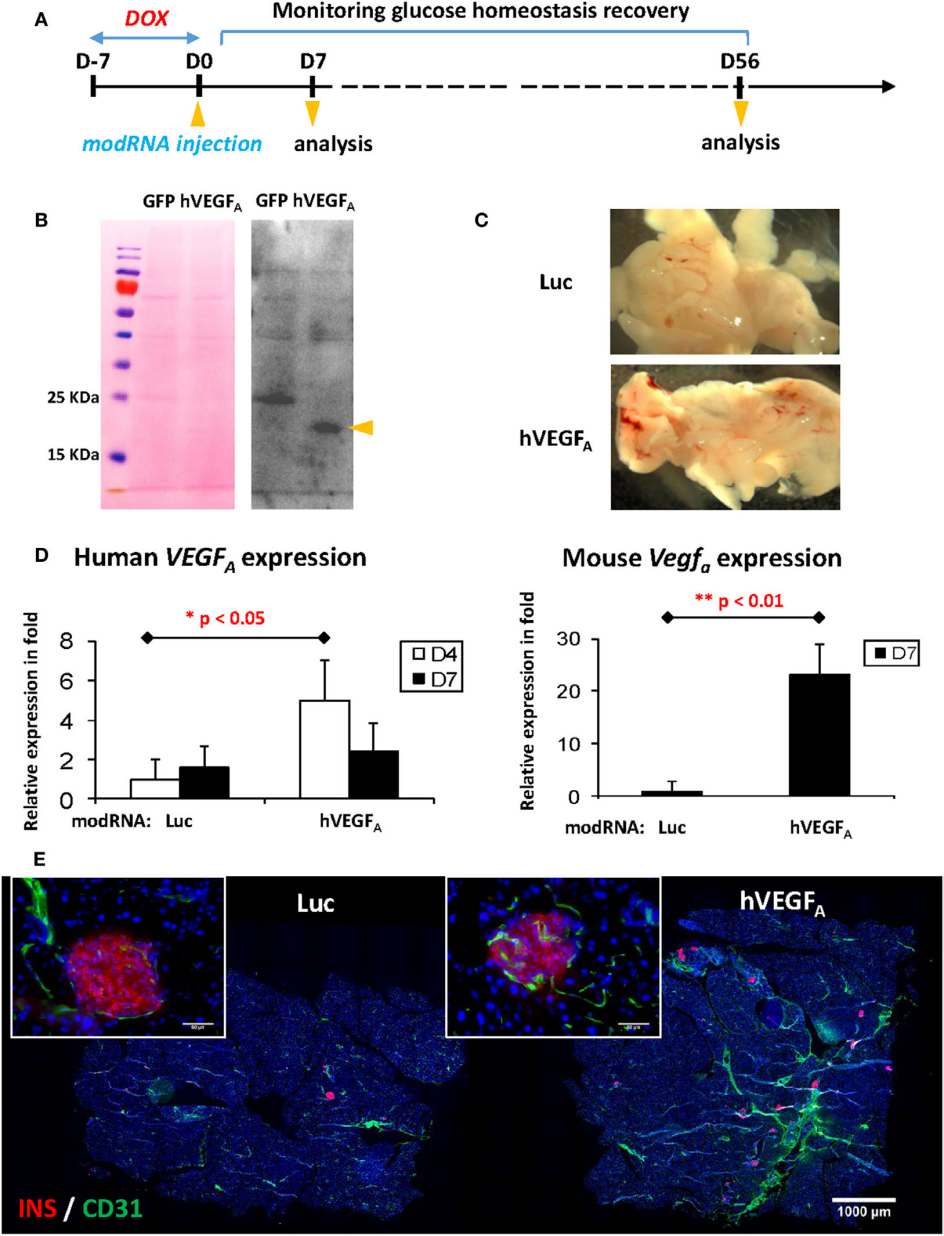
Experimental design and verification of human VEGF_A_ (hVEGF_A_) modified mRNA (modRNA) expression. **(A)** Experiment flow chart. **(B)** Expression of hVEGF_A_ protein by hESC transfected with hVEGF_A_ modRNA. hESC cultured in 6-well plate was transfected with 1 µg hVEGF_A_ or GFP modRNA (as control) per well. Culture supernatant was collected 24–48 h post-transfection, precipitated by trichloroacetic acid method, and blotted with hVEGF_A_ antibody. Arrow head points at hVEGF_A_ band, around 18–25 kDa. Ponceau S staining of the membrane before Western blot is shown as loading control. **(C)** Representative gross morphology of pancreas dissected from mice at D7 post-injection [luciferase (Luc) modRNA as control]. **(D)** qPCR for human and mouse VEGF_A_ expression in mouse pancreas at D4 and D7 post-injection (values are mean + SEM). **(E)** Representative insulin (red) and Cd31 (green) co-staining of pancreas at D56 post-injection (bar, 1,000 µm). Inserts are islets at high magnification (bar, 50 µm).

Having optimized the DOX treatment protocol, we then tested if transient expression of VEGF_A_ by local injection of modRNA could facilitate β cell regeneration with functional recovery. Schematic diagram of experiments was illustrated in Figure 3A. We first confirmed efficient translation of modRNA by transfecting 1 µg eGFP modRNA into hESCs *in vitro* (Figure S1 in Supplementary Material); and then confirmed hVEGF_A_ protein secretion by transfecting 1 µg control eGFP or hVEGF_A_ modRNA into hESCs *in vitro*. We collected supernatant 24−48 h post-transfection, and the secreted VEGF_A_ protein was examined by Western blot (Figure 3B). We then performed *in vivo* transfection in which βDTA mice were treated with 50 µg/mL DOX in drinking water for 7 days followed by intrapancreatic injection of 100 µg control luciferase (Luc) or hVEGF_A_ modRNA. Hyperglycemia was confirmed with the fed glucose level ≥15 mM or 270 mg/dL 7 days after DOX withdrawal. Mice were then assigned into the Luc or hVEGF_A_ group with each group had a similar mean fed glucose levels. Seven days after modRNA injection, we observed increased vascularization in the pancreas (Figure 3C) with expression levels of both the human and mouse VEGF_A_ mRNA significantly increased, respectively, in the hVEGF_A_ modRNA-treated group compared to that of the Luc control group (Figure 3D). Moreover, immunostaining for CD31 also showed that there was increased pancreatic vascularization in the hVEGF_A_ modRNA group 56 days post-transfection, and CD31^+^ blood vessels were found in close proximity to the pancreatic islets (Figure 3E). Our results showed that intrapancreatic injection of hVEGF_A_ modRNA induced transient expression of the hVEGF_A_ protein that promoted angiogenesis in the pancreas.

**Figure 4:**
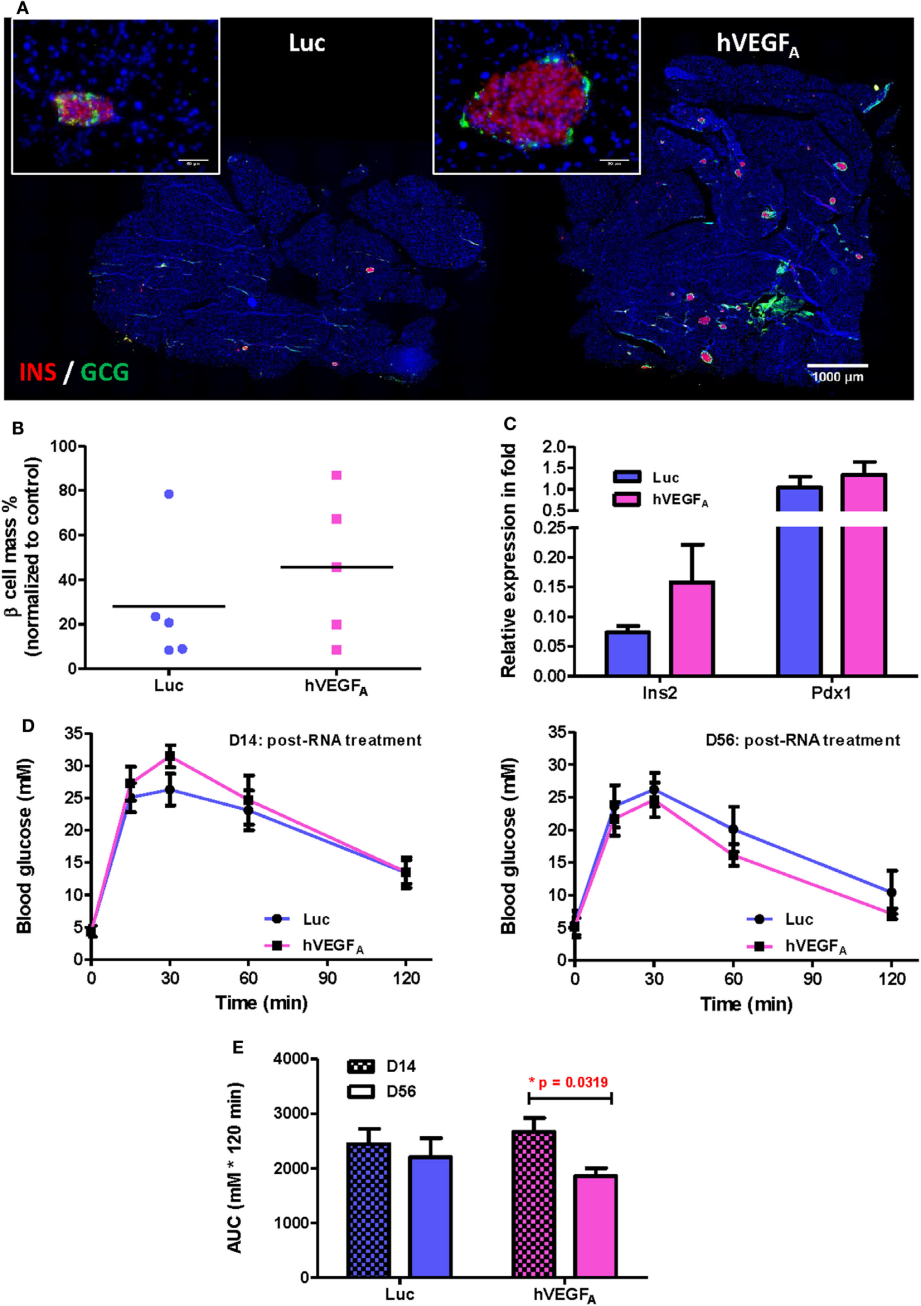
A single intrapancreatic human VEGF_A_ (hVEGF_A_) modified mRNA (modRNA) injection had only marginal improvement on β cell functional recovery. **(A)** Representative insulin (red) and glucagon (green) co-staining of pancreas at D56 post-injection (bar, 1,000 µm). Inserts are islets at high magnification (bar, 50 µm). **(B)** % β Cell mass of individuals at D56 post-injection (normalized to mean of non-treated controls, horizontal lines represent group means). **(C)** qPCR for Insulin (*Ins2*) and *Pdx1* expression at D56 post-injection (normalized to mean of non-treated controls). **(D)** Comparison of glucose tolerance test (GTT) (i.p.) between hVEGF_A_ and luciferase (Luc) (control) groups at D14 and D56 post-injection. **(E)** Analysis of area under curve of GTT results in panel **(D)**. *p = 0.0319 by paired *t*-test. Luc (luciferase) modRNA-treated mice as experimental control group, age-matched male βDTA mice without any treatment as non-treated controls for normalization, values are mean ± SEM, *n* = 5 per group.

To examine the morphology of pancreatic islets at day 56 post-transfection, we performed immunostaining for INSULIN (INS) and GLUCAGON (GCG) and found that the islets of the hVEGF_A_ modRNA-treated group restored their typical morphology with the INS^+^ β cells surrounded by the GCG^+^ α cells in the periphery (Figure 4A). Moreover, the hVEGF_A_ modRNA-treated group had increased number of islets (Figure 4A), improved β cell mass (Figure 4B), and increased insulin gene expression as determined by qPCR (Figure 4C) compared to that of the Luc modRNA-treated group. Furthermore, we performed GTT at days 14 and 56 post-transfection (Figure 4D), our results showed that glucose tolerance of the hVEGF_A_-treated group was significantly improved from D14 to D56; whereas the control group did not show any improvement during the same period (Figures 4D,E).

### Subtle Difference in Initial Drinking Behavior Contributes to a Large Variation in the Capacity of Individual βDTA Mouse for Spontaneous β Cell Regeneration

Despite treatment with a single dose of hVEGF_A_ modRNA improved glucose tolerance two months after β cell death (Figure 4E), there was a large variation in the recovery of β cell mass among individuals within the same group (Figure 4B). Since we found a strong positive correlation between the change in fed blood glucose level and accumulated DOX intake (Figure 1C), we hypothesized that polydipsia developed during the course of DOX treatment amplified the initial variation in daily water consumption and, therefore, the regenerative capacity among individuals within the same group. In fact, a large variation in the fasting blood glucose levels was also observed in βDTA mice treated with higher doses of DOX (200–1,000 µg/mL). Such a variation also increased with time after DOX withdrawal, suggesting a differential β cell regenerative capacity among individuals treated with the same dose of DOX (Figures S2B–D in Supplementary Material).

**Figure 5:**
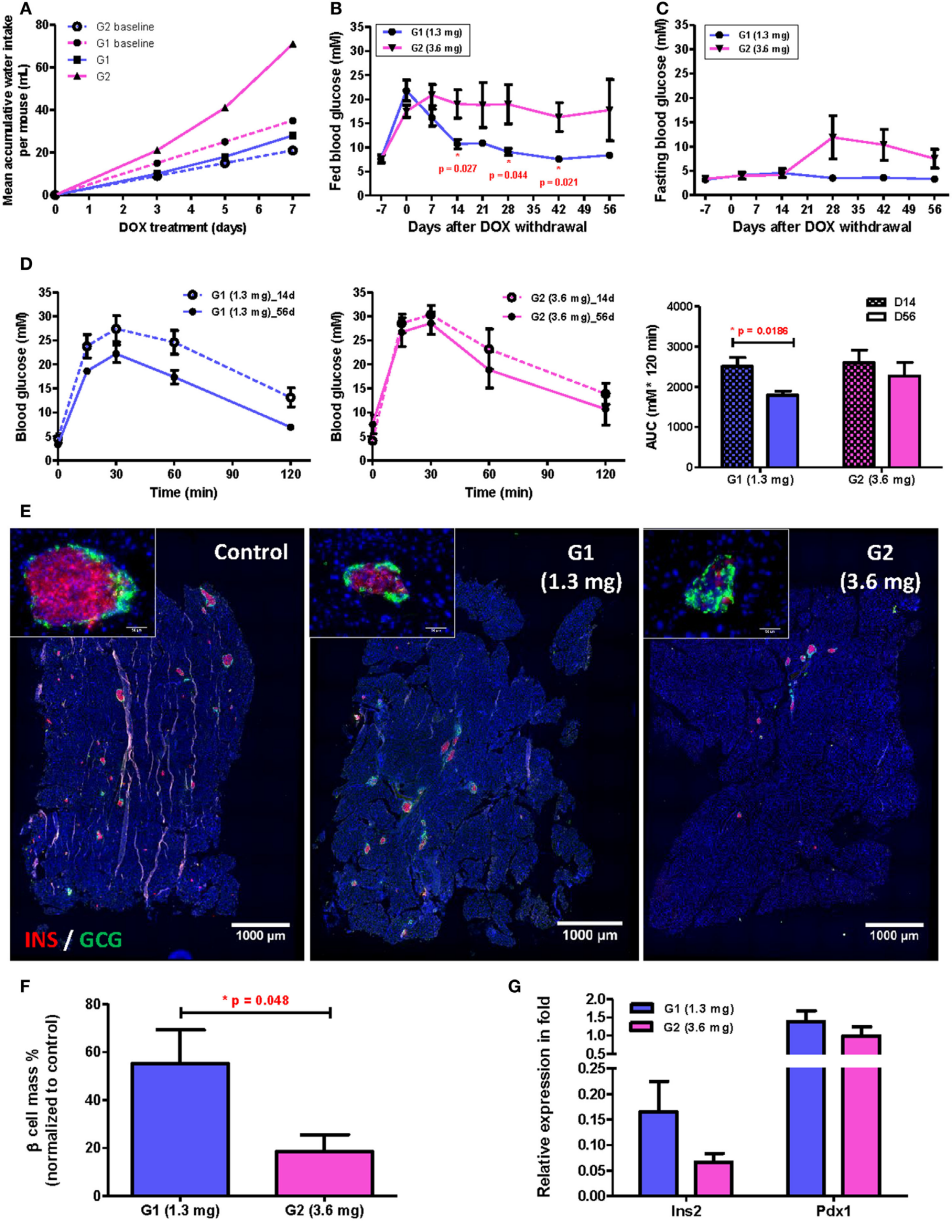
Subtle difference in drinking behavior has significant impact on β cell recovery among individuals after treating with 50 µg/mL doxycycline (DOX) for 7 days. **(A)** Comparison between mean expected (dashed lines) and actual (solid lines) accumulative DOX intake per mouse during treatment for subpopulations (G1 and G2) with different baseline daily water consumption. Each data point represents mean value of five individuals. **(B,C)** Fed and fasting glucose of two subpopulations before and after DOX treatment (*p < 0.05 by independent *t*-test comparing two groups at each time point). **(D)** Glucose tolerance test (i.p.) and area under curve analysis for two subpopulations at D14 and D56 after DOX withdrawal (*p = 0.0186 by independent *t*-test). **(E)** Representative insulin (red) and glucagon (green) co-staining of pancreas at D56 after DOX withdrawal (bar, 1,000 µm). Inserts are islets at high magnification (bar, 50 µm). **(F)** Comparison of % β cell mass (normalized to untreated controls) between two subpopulations at D56 after DOX withdrawal (*p = 0.048 by independent *t*-test). **(G)** qPCR for Insulin (*Ins2*) and *Pdx1* expression at D56 after DOX withdrawal (normalized to mean of non-treated controls). Values are mean ± SEM, *n* = 5 per group.

To reveal the influence of drinking habit on individual’s β cell regenerative capacity, we monitored the long-term β cell recovery for two groups of mice with subtle difference in their mean daily water consumed before treatment of 50 µg/mL DOX: the mean daily water consumption for these two groups were 3 mL (group/G1, *n* = 5) and 5 mL (group/G2, *n* = 5) per mouse, respectively; and both were within the normal physiological range. During DOX treatment, polydipsia developed in individual mouse at a different speed according to individual’s initial daily water consumption, so the difference in accumulated DOX intake between the two groups was dramatically amplified (as illustrated by the dashed and solid lines representing expected and actual intake, respectively, in Figure 5A). At the end of 7-day treatment, the mean accumulated DOX intake for individuals of G1 was 1.3 mg and of G2 was 3.6 mg, respectively. Furthermore, despite the two groups showed very similar fed (Figure 5B, day −7) and fasting (Figure 5C, day −7) blood glucose levels before treatment, a significant difference in their fed glucose level was observed since day 14 after DOX withdrawal (Figure 5B). Therefore, a slight difference in the drinking habit among individuals treated with the same concentration of DOX water led to significant difference in the accumulated DOX intake that was evidenced by neither the fed (Figure 5B, day 0) nor fasting (Figure 5C, day 0) blood glucose level at the time when DOX was withdrawn.

In fact, we also observed a significant difference in β cell functional recovery with respect to accumulated DOX intake even within the range of 1.3−3.6 mg per mouse. β Cell function had been recovering in G1 mice as evidenced by the continued decrease in both fed (Figure 5B) and fasting (Figure 5C) blood glucose levels with time; and the reduced individual variation in both fed and fasting blood glucose levels with time. Moreover, the G1 group also demonstrated a significantly improved glucose tolerance by comparing results of GTT at D56 and D14 post DOX treatment (Figure 5D). Immunostaining for INS and GCG also showed that the islet morphology of the G1 was better than the G2 group at D56 post DOX treatment (Figure 5E). There were also more INS^+^ β cells (Figure 5E), significantly greater β cell mass (Figure 5F) and higher *Insulin* gene expression (Figure 5G) in the G1 than G2 group. Together, our results showed that long-term recovery of β cell mass and glucose tolerance was dependent on the accumulated DOX intake during the initial treatment phase; and individual variation in the rate of β cell regeneration was significantly influenced by one’s drinking habit even if the same concentration of DOX was given to the group in the same bottle of drinking water.

### The Therapeutic Efficacy of hVEGF_A_ modRNA on β Cell Regeneration Is Dependent on the Degree of Initial β Cell Loss

Because hVEGF_A_ modRNA demonstrated some improvement in glucose tolerance following DOX-induced β cell death; yet there was large variation in β cell mass among individuals within the same group (Figure 4), and we found that the difference in accumulated DOX intake also contributed to variation in spontaneous β cell recovery (Figure 5), we hypothesized that the effect of hVEGF_A_ modRNA treatment might also be dependent on the accumulated DOX intake. To address this, two cohorts with slightly different drinking habit were treated with 50 µg/mL DOX in drinking water for 7 days, and the mean accumulated DOX intake by each mouse at the end of treatment was 1.5 and 3.0 mg, respectively. In each cohort, mice were assigned to balanced control (Luc modRNA) and treatment (hVEGF_A_ modRNA) groups based on comparable fed blood glucose levels at day 0. Experiments were conducted as outlined in Figure 3A.

**Figure 6:**
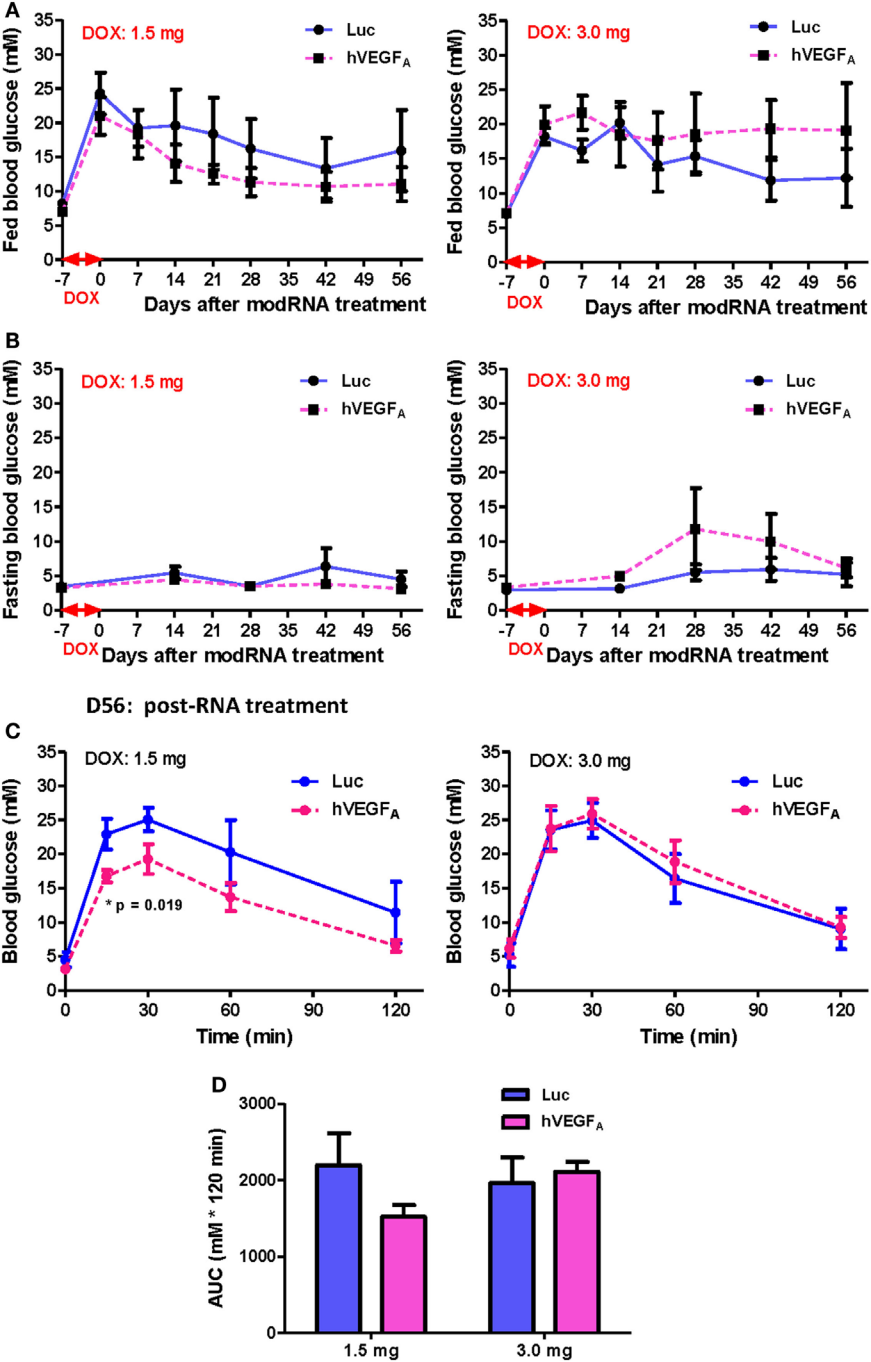
The effect of human VEGF_A_ (hVEGF_A_) modified mRNA (modRNA) on β cell functional recovery depends on the accumulative doxycycline (DOX) intake during DOX treatment. **(A,B)** hVEGF_A_ modRNA had different effects on the fed and fasting glucose kinetics for cohorts receiving 1.5 and 3.0 mg DOX per mouse. **(C,D)** hVEGF_A_ modRNA had different effects on the glucose tolerance recovery at D56 post-modRNA injection for cohorts receiving 1.5 and 3.0 mg DOX per mouse (*p = 0.019 by independent *t*-test comparing two groups at each time point). Values are mean ± SEM. For the cohort receiving 1.5 mg DOX per mouse, *n* = 4 for luciferase (Luc) control group and *n* = 6 for hVEGF_A_ treatment group. For the cohort receiving 3.0 mg DOX per mouse, *n* = 6 for Luc control group and *n* = 4 for hVEGF_A_ treatment group.

After injection of 100 µg modRNA, the fed (Figure 6A) and fasting (Figure 6B) blood glucose level of the hVEGF_A_ modRNA-treated group decreased more rapidly than that of the Luc group in the cohort received 1.5 mg DOX accumulatively from day −7 to 0. However, a reverse trend was observed in the cohort received 3.0 mg DOX (Figures 6A,B). It is of noted that the DOX dose 1.5 mg did not result in impaired fasting but fed blood glucose level throughout the experiment; it was likely that less insulin is needed to maintain fasting than fed blood glucose levels. Furthermore, our GTT results also demonstrated that the hVEGF_A_ modRNA treatment improved glucose tolerance in the cohort received 1.5 mg DOX (Figures 6C,D); while such effect was not observed in the cohort received 3.0 mg DOX (Figures 6C,D). Therefore, the benefit of hVEGF_A_ modRNA in β cell regeneration was observed only in individuals with less severe β cell loss as evidenced by better improved glucose tolerance in βDTA mice received lower (1.5 mg) but not higher (3.0 mg) amount of DOX.

## Discussion

In this study, we aimed to establish a model for evaluating the therapeutic potential of VEGF_A_ modRNA in promoting β cell functional recovery after DOX-induced β cell death using the βDTA mice. Since the C57 background appears to be more sensitive to DOX induction compared to its parental line (17, 21), a relatively lower dose of DOX (50 µg/mL) was used to allow some degree of spontaneous β cell recovery within a reasonable period of time. Nevertheless, the relationship between VEGF_A_ and β cells has not been straightforward (2). It has been reported that VEGF_A_ induces angiogenesis and promotes proliferation of the early pancreatic progenitors; whereas mature blood vessels restrict their further differentiation and maturation into the endocrine lineage during development (1, 22). In adult pancreas, sustained overexpression of VEGF_A_ induces an inflammatory microenvironment for islets (11, 23); but transient expression of VEGF_A_ promotes β cell regeneration and improves β cell function after injury (12, 13, 24). Therefore, in this study, we attempted to utilize the transient nature of chemically modRNA and examine the beneficial effect of VEGF_A_ modRNA in β cell regeneration after injury. Previous reports have demonstrated that gene expression is transient yet highly efficient following modRNA transfection (15, 25, 26), so it has been used as a therapeutic delivery agent in many studies (27–29). We have also successfully applied hVEGF_A_ modRNA in promoting murine cardiac regeneration after myocardial infarction (14, 16).

To our surprise, however, there was only some marginal effect by hVEGF_A_ modRNA in improving β cell mass and glucose tolerance recovery after injury despite transient expression of VEGF_A_ should promote β cell regeneration as previously demonstrated (12, 13). Importantly, we found very large individual variation within the control and treatment groups although the group assignment ensured that individual blood glucose level was balanced before modRNA treatment. We also suspect that large variation within groups would lead to misinterpretation of data. Therefore, we seek to investigate the cause of variation in the same cage of mice treated under the same condition.

In conventional protocols, DOX is administrated through drinking water to induce gene expression in transgenic mice *via* the TET-rtTA system (18, 30). However, when β cell death and hyperglycemia became more severe, βDTA mice could develop polydipsia before DOX withdrawal, increasing the total DOX intake disproportionately. As a result, in addition to the different degree of innate β cell regenerative capacity, the degree of initial β cell lesion could vary among individuals. Although absorption, metabolism, clearance of DOX, and sensitivity of the TET-rtTA genetic switch could contribute to variation in β cell lesion, the actual DOX intake was the most direct contributor when DOX is administrated *via* drinking water. In this scenario, even subtle difference in drinking habit might lead to differentially accumulated DOX intake, contributing to large variation in long-term β cell functional recovery among individuals.

To address this hypothesis, we monitored two groups of mice with slight difference in drinking habit but within a normal physiological range. Even being treated with 50 µg/mL DOX for 7 days, the relative difference in accumulative DOX intake became large (1.5 vs 3.0 mg). Although both fed and fasting blood glucose remained similar until 14 days after DOX withdrawal, the course of β cell functional recovery was significantly different between these two groups. When mice are treated with DOX water at the same concentration, the absolute difference in actual DOX intake is relatively small and not reflected by fasting or fed blood glucose immediately, but the degree of β cell lesion may vary with time among individuals given the difference in drinking habit. Based on our observations, it may take at least 14 days after DOX withdrawal to reveal the effect of difference in accumulative DOX dose on fed glucose for a low dose of DOX at 50 µg/mL, but β cell recovery may have already initiated during this time. Furthermore, it is generally preferred to administer therapeutic agents as early as possible after β cell lesion so protections can be provided before β cell death (12, 13). As a result, variation in actual DOX intake cannot be controlled by balancing individuals in group assignment based on their blood glucose levels before testing therapeutic agents.

Furthermore, the individual variation in long-term β cell functional recovery is not eliminated by increasing DOX dose for a “saturated” β cell death. We followed a cohort of mice treated with 2,000 µg/mL DOX and β cell functional recovery was not observed 63 days after treatment at this high dose (Figure S3E in Supplementary Material). Under this dose for 7 days, the accumulated DOX intake by a mouse should be at least 42 mg (assuming constant minimal water consumption at 3 mL/day). By retrospectively splitting a large cohort into two balanced groups with almost identical fed and fasting glucose at D0 posttreatment, the blood glucose became significantly different during the course of recovery (Figures S3A,B in Supplementary Material). The variation in fasting glucose among individuals in the entire cohort became progressively large after DOX withdrawal, assuming a bimodal distribution such that the difference between two groups became significant at D49 (Figure S3C in Supplementary Material). In this case, it is likely that the degree of initial β cell death was different between these two groups despite the different regenerative capacities, and difference in drinking habit between groups can resulted in huge difference in total DOX intake especially at high doses. It is important to note that the conventional portable glucometer fails to reveal the actual blood glucose difference beyond its detection limit (≤33.3 mM or 600 mg/dL), resulting in an underestimated “saturation” of DOX dose.

Apparently, the great individual variation in β cell recovery is an infirmity inherited to this DOX-induced β cell lesion model. We have identified three embedded characteristics that could contribute to such infirmity: the high sensitivity of the TET-rtTA system, the positive feedback relationship between β cell death (hyperglycemia and polydipsia) and DOX intake, and a potential variation in the degree of initial β cell lesion among individuals shortly after DOX withdrawal. The sensitivity and dose dependence of the TET-rtTA system combined with the ease of DOX administration through drinking water have been exploited by some reporter mouse lines (19). Since gene expression induced by DOX in those models does not alter drinking habit, induction of gene expression can be easily manipulated by DOX concentration and treatment duration. However, β cell death induced by DOX results in polydipsia that compromises the adjustability of β cell lesion with DOX concentration when DOX is supplied in drinking water. Although the same DOX administration method was adopted in other studies using βDTA mice (17, 21), those studies all focused on spontaneous β cell regeneration after β cell injury over time. In those longitudinal studies, physiological parameters of the same animal were pair-wisely compared across different time points during recovery or compared with untreated healthy control mice, but the DOX-treated mice were not split into different experimental groups for comparison (17, 20, 21). In contrast, it is necessary to generate relatively homogenous cohorts in terms of β cell lesion as a model to evaluate therapeutic agents with the potential in promoting β cell functional recovery. Nevertheless, whether more controlled administration of DOX, for instance, *via* oral gavage, could reduce individual variation during spontaneous and acquired beta cell regeneration awaits further investigations.

Altogether, our results demonstrated some degree of improved beta cell mass and function posttreatment of hVEGF_A_ modRNA in the DOX-induced β cell death model. Since hVEGF_A_ modRNA harnesses great therapeutic potential and drug company including AstraZeneca has already filed clinical trial application with an attempt to use VEGF_A_ modRNA in treating cardiovascular diseases, our results might foster future development of VEGF_A_ modRNA for treatment of other diseases including diabetes. Nevertheless, the beneficial effects of hVEGF_A_ modRNA appeared to be sensitive to the actual DOX intake, implying its effect is dependent on the degree of β cell damage. Our results also highlight the caution in interpretation of β cell regeneration data given drinking habit contributed variation in the degree of initial β cell damage particularly using this βDTA mouse model.

## Materials and Methods

### Mice and DOX Treatment

The βDTA mice [Tg(Ins2-rtTA)2Efr Tg(teto-DTA)1Gfi/J] were purchased from The Jackson Laboratory and maintained in the Laboratory Animal Center of The Chinese University of Hong Kong (CUHK). All experimental procedures were approved by the Animal Experimentation Ethics Committee of CUHK and performed in compliance with “Guide for the Care and Use of Laboratory Animals” (8th edition, 2011) established by National Institutes of Health. Male mice of 8−12 weeks were used for experiments, and there was no hyperglycemia, glucose intolerance, or polydipsia observed before DOX induction. To induce β cell death, three to five mice were housed per cage with free access to drinking water contained 50−2,000 μg/mL DOX (Sigma, D9891) for 7 days. Water bottles were protected from light, and DOX water was frequently checked for replacement during treatment. One week before DOX treatment, the average daily water consumption per cage over 7 days was measured by marking the calibration on water bottles, and the baseline mean daily water consumption per mouse in a cage was estimated based on the number of mice housed. During DOX treatment, the mean accumulative DOX intake per mouse within a period was estimated as (total water consumption per cage × doxycycline concentration)/number of mice housed.

### Blood Glucose Monitoring and GTT

Blood samples were collected from tail vein and measured by a commercial glucometer (Contour^®^ TS, Bayer). The fed blood glucose levels were measured regularly at 9:00−12:00; and the fasting blood glucose levels were measured after an overnight fasting for 14−16 h. For GTT, mice were injected intraperitoneally (i.p.) with 2 g glucose per kilogram body weight after taking the fasting blood glucose levels; and the blood glucose levels were monitored at 15, 30, 60, and 120 min after glucose injection. The blood glucose readings were then plotted against time after glucose injection, and the area under curve (AUC) was calculated using GraphPad Prism 5.0. The baseline adjusted AUC was used sometimes if the fasting blood glucose levels between groups were significantly different.

### Synthesis of modRNA and *In Vivo* Intrapancreatic Delivery

Chemically modRNA for eGFP, Luc and hVEGF_A_ were synthesized as previously described (15, 16, 31). After DOX withdrawal, 100 µg hVEGF_A_ or Luc modRNA was injected into the mouse pancreatic tail directly in 60 µL mixture containing 10 µL modRNA, 10 µL Opti-MEM I (Thermo Fischer, 31985), and 40 µL Lipofectamine RNAiMAX (Thermo Fischer, 56532), using a 29G insulin syringe (BD, 320431) as previously described (32).

### hESC Cultures, *In Vitro* modRNA Transfection, and Protein Expression Analysis

The human embryonic stem cell (ESC) line was maintained as previously described (15). 1 µg hVEGF_A_ or eGFP modRNA was transfected per well of cells cultured on 6-well plates using Lipofectamine RNAiMAX as previously described (15, 31). Transfection medium was replaced by conventional culture medium 6 h after transfection. During 24–48 h post-transfection, cells were cultured in fresh serum free medium. The culture supernatant was collected afterward, and proteins were precipitated by the trichloroacetic acid method. The presence of hVEGF_A_ protein was confirmed by Western blot (mouse anti-hVEGF_A_ antibody, 1/100, BD Catalog no. 554359). The membrane was lightly stained by Ponceau S as loading control before blotting with antibody.

### RNA Extraction and qPCR Analysis

The mouse pancreata were dissociated in TRIzol reagent, and total RNA was isolated according to manufacturer’s instruction (Thermo Fischer, 15596018). cDNA was synthesized using iScript cDNA synthesis kit (BioRad, 170-8891), and qPCR was performed using iTaq universal SYBR Green supermix (BioRad, 172-5120) on QuantStudio 12K Flex Real-Time PCR system (Thermo Fischer). The relative gene expression level of each sample was calculated by 2^−ΔΔCT^ method using mouse β-*actin* or human *GAPDH* as internal control. hESC cDNA and pancreatic cDNA of age-matched male βDTA mice (untreated) or mouse ESC cDNA were used as calibrator to normalize gene expression for hESC samples and mouse pancreatic samples, respectively. Primers used for mouse genes: *Vegfa* (5′-GCTTCCTACAGCACAGCAGA-3′; 5′-AATGCTTTCTCCGCTCTGAA-3′), *Ins2* (5′-GGAGCGTGGCTTCTTCTACA-3′; 5′-CAGTGCCAAGGTCTGAAGGT-3′), *Pdx1* (5′-GATGAAATCCACCAAAGCTCACGC-3′; 5′-AATTCCTTCTCCAGCTCCAGCAGC-3′), and β-*actin* (5′-TTTGCAGCTCCTTCGTTGCCG-3′; 5′-TTTGCACATGCCGGAGCCGTT-3′). For human genes: *VEGFA* (5′-AAGGAGGAGGGCAGAATCAT-3′; 5′-CCAGGCCCTCGTCATTG-3′) and *GAPDH* (5′-TGTTGCCATCAATGACCCCTT-3′; 5′-CTCCACGACGTACTCAGCG-3′).

### Immunohistochemistry and Image Analysis

The wet weights of mouse pancreata were taken immediately after dissection. Tissues were fixed in 4% PFA at 4°C overnight, washed three times by PBS, and infiltrated by 30% sucrose at 4°C. The pancreata were frozen in OCT blocks and sectioned at 8 µm thickness. Discontinued sections with 15 sections apart were mounted onto a slide. For quantification of each marker, 6 discontinued sections were stained and sampled per mouse pancreas. Immunofluorescence was performed using the following primary antibodies: guinea pig anti-insulin (1/400, Dako, A0564), rabbit anti-glucagon (1/200, Dako, A0565), and rat anti-CD31 (1/100, BioLegend, 102501). AlexaFluor-488 or 546 linked goat secondary antibodies were used (1/500, Thermo Fischer), and nuclei were stained by Hoechst 33342 (1 µg/mL, Thermo Fischer, H3570) before mounted with Aqua-Poly/Mount (Polysciences, 18606). For image acquisition, each entire pancreas section was scanned, and images were automatically stitched using Leica fluorescence microscope with LAS AF software (Leica DM4000, Germany). Quantitative image analysis was performed using Image J (NIH). The β cell mass was calculated as followed (13, 17): pancreas wet weight × (the sum of INSULIN^+^ stain area of 6 discontinued sections)/(the sum of total nucleated area of 6 discontinued sections) × 100%. For comparison, the β cell mass of each mouse in the experimental groups was normalized to the mean value of β cell mass of age-matched male βDTA mice (without any treatment) and presented in percentage.

### Statistics

Data analysis was conducted using GraphPad Prism 5.0. To compare two independent groups, independent *t*-test was performed, and one-way ANOVA followed by Bonferroni’s or Dunnett’s multiple comparison was used for more than two independent groups. Paired *t*-test was used to compare the same group at the indicated time points for longitudinal study. Pearson correlation coefficient *r* was calculated to measure correlation. *p* Values were specified for each test, and a *p* value < 0.05 was considered statistically significant.

## Ethics Statement

The mice were maintained in the Laboratory Animal Center of The Chinese University of Hong Kong (CUHK). All experimental procedures were approved by the Animal Experimentation Ethics Committee of CUHK and performed in compliance with “Guide for the Care and Use of Laboratory Animals” (8th edition, 2011) established by National Institutes of Health.

## Author Contributions

SL and JL performed experiments and analyzed data; KL designed experiments and analyzed data; SL and KL wrote the manuscript.

## Conflict of Interest Statement

The authors declare that the research was conducted in the absence of any commercial or financial relationships that could be construed as a potential conflict of interest.

## Supplementary Material

Figure S1 GFP expression in hESC transfected with eGFP modified mRNA (modRNA) after 48 h. hESCs cultured in 6-well plate were transfected with 1 µg eGFP modRNA or vehicle control (RNAimax only). After 48 h, green auto-fluorescence was only observed in eGFP modRNA-treated cells **(A)** but not in control cells **(B)**. Scale bar, 50 µm.

Figure S2 Mice treated with doxycycline (DOX) at a dose of 200 µg/mL or above for 7 days did not recover in blood glucose control 63 days after DOX withdrawal. **(A)** Fasting blood glucose kinetics for different groups after DOX withdrawal. Values are mean ± SEM, *n* = 4 per group. **(B–D)** Dot plots to reveal individual variation in fasting glucose for different groups at each time point.

Figure S3 Mice treated with 2,000 µg/mL doxycycline (DOX) for 7 days did not recover in glucose tolerance 63 days after DOX withdrawal but demonstrated progressively increased individual variation and bimodal sample stratification in blood glucose. Ten mice underwent DOX treatment was monitored after DOX withdrawal. After data collection, they were retrospectively split into two artificial groups G1 and G2 (*n* = 5 per group) based on almost identical fed and fasting glucose on the day of DOX withdrawal. **(A,B)** Fed and fasting glucose kinetics of two artificial groups after DOX withdrawal (**p* < 0.05 by independent *t*-test comparing two groups at each time point, values are mean ± SEM). **(C)** Dot plots to illustrate progressively increasing individual variation and bimodal stratification of the entire cohort (artificial group means as horizontal lines). **(D,E)** Glucose tolerance of both artificial groups continued to deteriorate from D35 to D63 after DOX withdrawal. **(D)** Glucose tolerance test (GTT) (i.p.). Values are mean ± SEM. **(E)** Area under curve analysis of GTT, values are mean + SEM, **p* = 0.028 and ****p* < 0.001 by paired *t*-test.
