# Supplementary material for: Individual Variation in Conditional β Cell Ablation Mice Contributes Significant Biases in Evaluating β Cell Functional Recovery
Source: Front Endocrinol (Lausanne). 2017 Sep 14;8:242. doi: 10.3389/fendo.2017.00242 (PMC5604075; doi:10.3389/fendo.2017.00242)
Supplement: Figure S1 — GFP expression in hESC transfected with eGFP modified mRNA (modRNA) after 48 h. hESCs cultured in 6-well plate were transfected with 1 µg eGFP modRNA or vehicle control (RNAimax only). After 48 h, green auto-fluorescence was only observed in eGFP modRNA-treated cells (A) but not in control cells (B). Scale bar, 50 µm. [file Presentation_1.PPTX]

## Slide 1
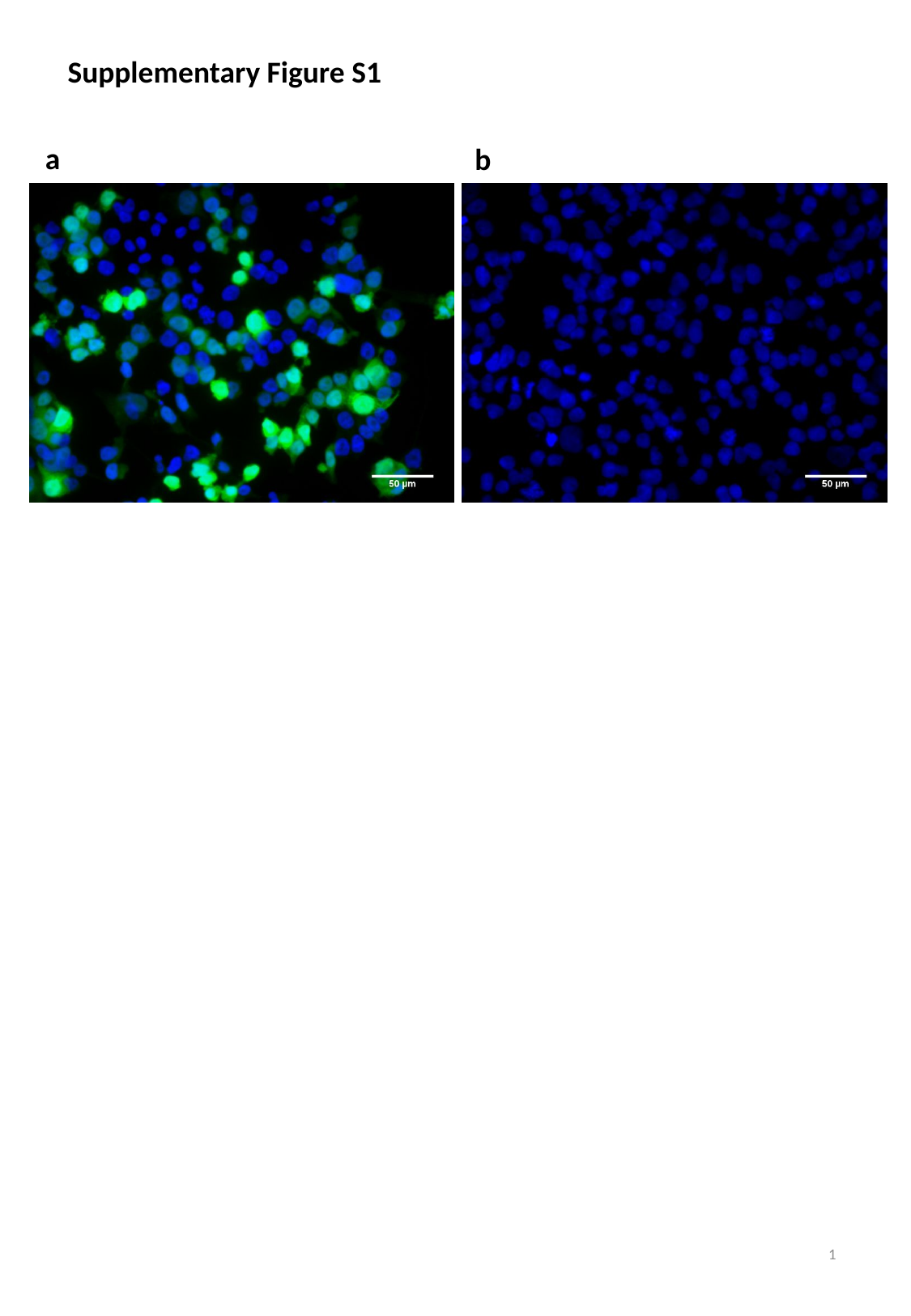

Supplementary Figure S1
a
b
1

## Slide 2
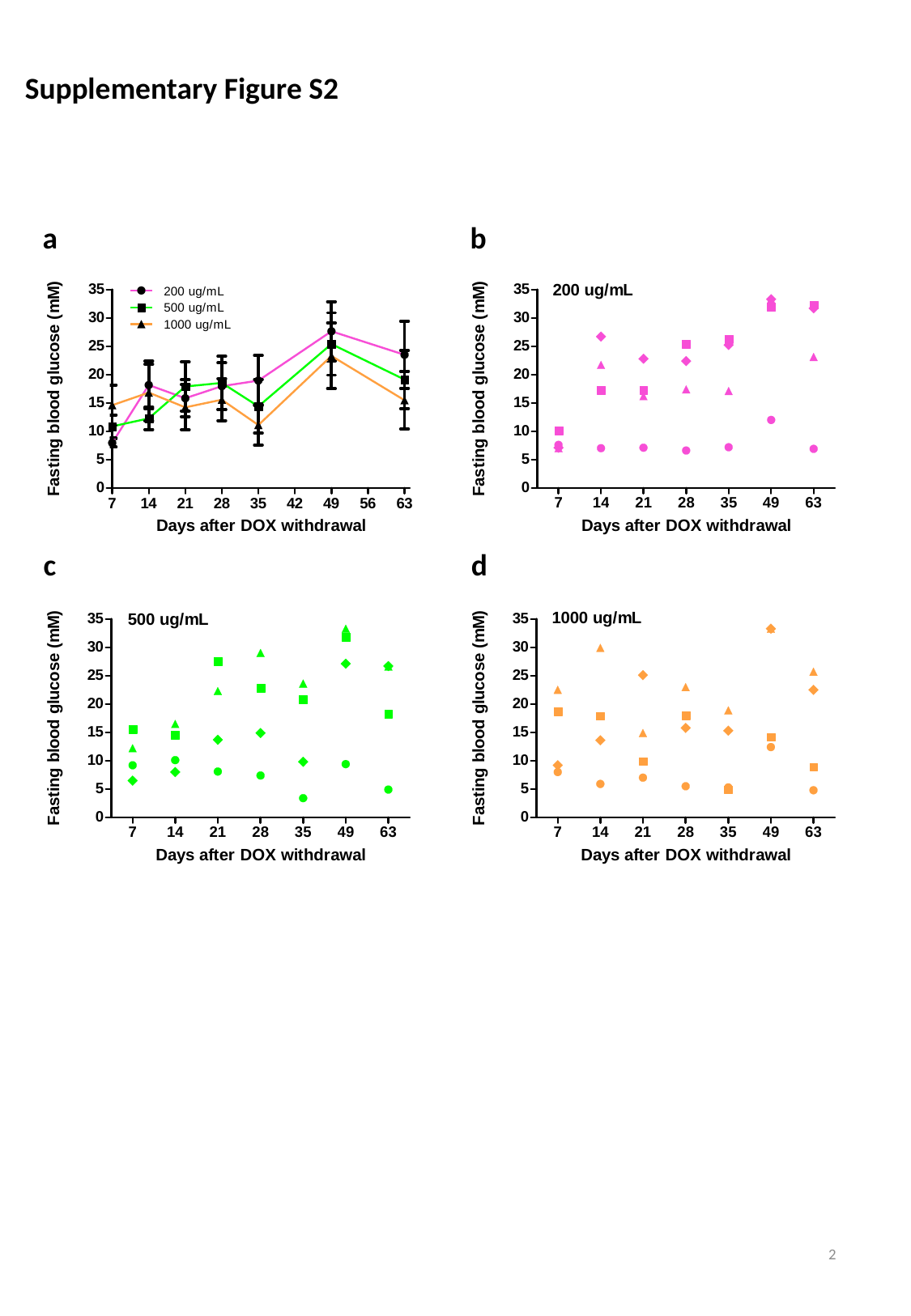

Supplementary Figure S2
a
b
c
d
2

## Slide 3
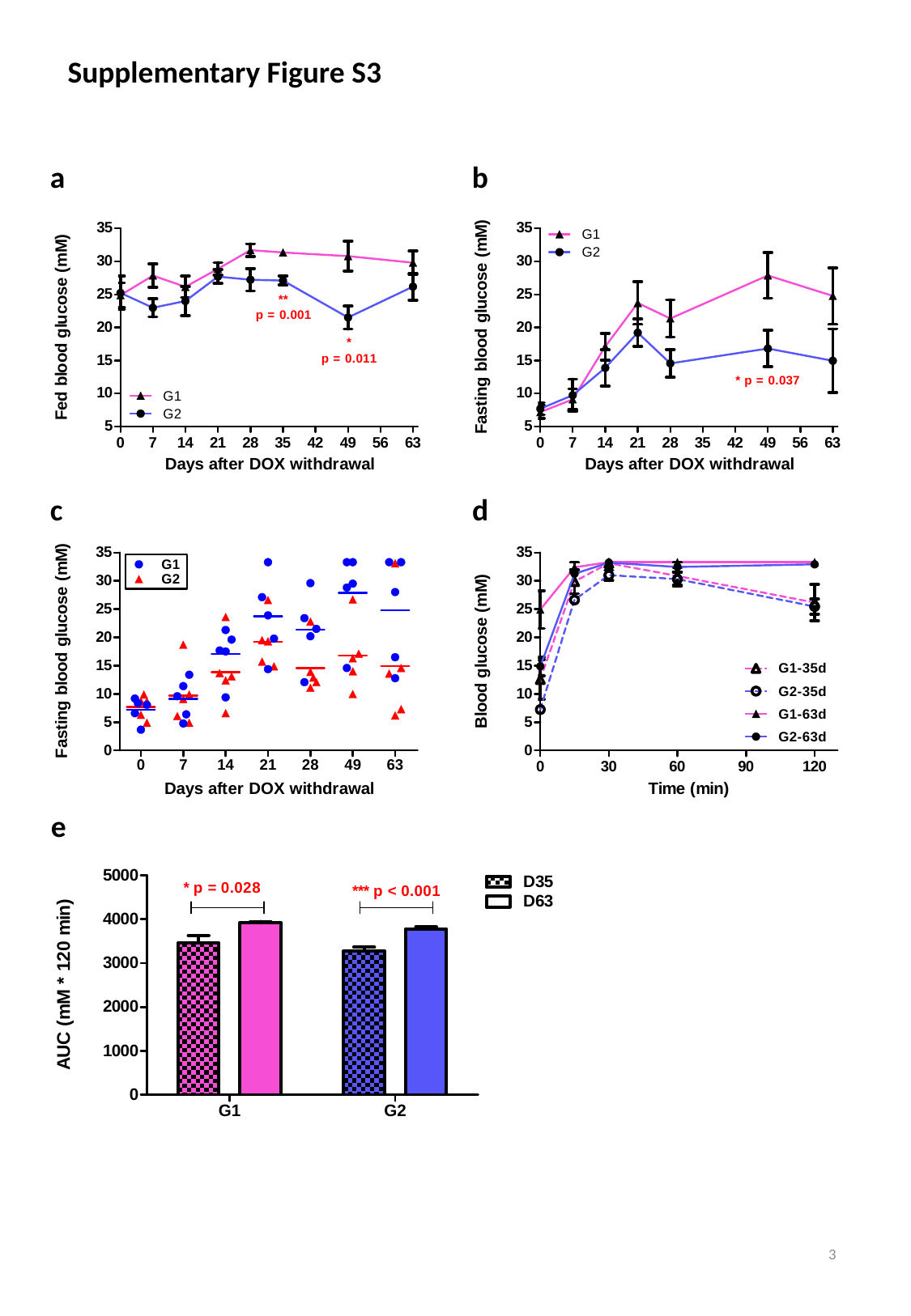

Supplementary Figure S3
a
b
c
d
e
3
